# Correlation of glycemic regulation and endotrophin in patients with type 2 Diabetes; pilot study

**DOI:** 10.1186/s13098-021-00628-5

**Published:** 2021-01-21

**Authors:** Sengul Aydin Yoldemir, Yucel Arman, Murat Akarsu, Ozgur Altun, Mustafa Ozcan, Tufan Tukek

**Affiliations:** 1 Internal Medicine Department, Okmeydanı Training and Research Hospital, Istanbul, Turkey; 2 Faculty of medicine, Internal Medicine Department, Istanbul University, Istanbul, Turkey

**Keywords:** Endotrophin, Type 2 diabetes mellitus, Nephropathy, Microalbuminuria

## Abstract

**Background:**

Endotrophin is one of the extracellular matrix proteins secreted by adipose tissue. In this study, we aimed to investigate the effects of changes in blood glucose levels on serum endotrophin levels secreted by adipose tissue and thus on diabetes.

**Methods:**

In this prospective pilot study included 78 patients with type 2 diabete (T2D) with hemoglobin A1c level > 9 %. Lifestyle changes were recommended and appropriate medical treatment was initiated to all patients in order to reach the target HbA1c level. Data of anthropometric measurements, urinary albumin creatinine ratio (UACR), serum lipid parameters and endotrophin were collected in patients; all examinations were repeated after 3 months. Analysis was performed using Paired-Samles T test and Spearman tests.

**Results:**

Of patients, 23 were female (54.8 %) and 19 were male (45.2 %). Mean age was 55.2 years, with mean diabetes age of 8.14 ± 5.35 years. After 3 months follow-up, HbA1c, fasting glucose, C-reactive protein(CRP), UACR and endotrophin levels were observed to clearly reduce. The variation in serum endotrophin levels examined at the start of the study and in the 3rd month was identified to have a positive correlation with the variation in HbA1c and UACR levels (r = 0.342, p = 0.02; r = 0.484, p = 0.001). Multiple linear regression analysis showed percentage variation values (δ)-endotrophin levels were only independently correlated with (δ)-UACR (model r^2^ = 0.257, p value = 0.00).

**Conclusions:**

Endotrophin levels decreased significantly with the decrease in HbA1c. Unexpectedly, this reduction in endotrophin levels is closely related to the decrease in UACR, regardless of blood glucose regulation. We think that studies targeting endotrophin will contribute to the diagnosis, treatment and follow-up of diabetic nephropathy in the future.

## Background

Type 2 diabetes mellitus (T2D) is a chronic degenerative metabolic disease affecting more than 425 million people in the world in general. By 2045 it is expected the number of people affected by diabetes will reach 629 million [1]. The World Health Organization (WHO) accepts both T2D and obesity as 21st century epidemic diseases [2].

Adipose tissue storing energy, it is an endocrine organ ensuring the secretion of a variety of adipokines regulating glucose and lipid metabolism. Collagen VI is one of the extracellular matrix (ECM) proteins secreted by adipose tissue. Endotrophin is the C-terminal cleavage product, involving C5 domain of COL6α3 [3, 4]. A variety of studies in mice and humans have associated systemic insulin resistance associated with obesity with endotrophin levels. High levels of serum endotrophin found in patients with insulin resistance (IR) [4–6].

There are many studies investigating the correlation between adipokines with IR and T2D development [7–10]. However, there is insufficient data about the endotrophin levels in patients with T2D. Additionally, the relationship between glycemic regulation and endotrophin level has not been researched. Our study primarily targeted glycemic control of patients with poor glycemic control with lifestyle changes and medical treatment and aimed to research the effect of blood glucose regulation on endotrophin level in patients with T2D. Secondaryly, we investigated the relationship of endotrophin with kidney function in patients with diabetes.

## Methods

Our study included 78 patients with T2D diagnosis according to American Diabetes Association (ADA) criteria [11], aged from 30 to 70 years with hemoglobin A1c level > 9 %. Patients with chronic kidney injury (GFR < 60 ml/min/1.73 m^2^), liver diseases, chronic inflammatory disease and malignancy, and patients using steroids, alcohol or drugs were excluded from the study. Additionally, to exclude patients with type 1 diabetes mellitus and latent autoimmune diabetes in adults (LADA), participants with c-peptide levels < 0.8 ng/mL were excluded from the study.

### Study design

This prospective study was carried out with patients who were not able to regulate blood glucose in the primary and secondy care hospitals and who were referred to the internal diseases outpatient clinics of our tertiary care hospital. Patients who met the inclusion criteria were included in the study. Our study was planned in accordance with the ethical standards of the Helsinki Declaration. All participants gave written informed consent after being informed about their treatment and laboratory tests. Before the study, ethics committee permission was obtained from the local ethics committee (June 2018 dated and numbered 988).

### Study protocol and treatment

The study included 78 patients. Due to incompliance with treatment and loss during follow-up, 11 patients were excluded. During the study duration target HbA1c values could not be reached for 25 patients and they were excluded from the study. On the first visit, height and weight measurements were performed, and body mass index (BMI=(weight/(height)²)) was calculated. HbA1c, fasting blood glucose (FBG), c-reactive protein (CRP), high density lipoprotein cholesterol (HDL-C), low density lipoprotein cholesterol (LDL-C), triglycerides, serum endotrophin and spot urinary albumin to creatinine ratio (UACR) were examined.

Patients were given personalized medical nutrition treatment (MNT) and an exercise program. MNT was prepared by a dietitian experienced in diabetes nutrition. Patients were recommended to obtain 30 % of daily energy requirements from fats (< 7 % from saturated fats) and 15–20 % from proteins. Patients who were overweight and obese had planned calorie intake regulation to ensure 5 % loss of weight in the first 3 months. Diet planning considered the patient’s lifestyle, education level and eating habits. Lifestyle changes suggested by ADA guides; ‘Most adults with type 1 and type 2 diabetes should engage in 150 minute or more of moderate to vigorous-intensity aerobic activity per week, spread over at least 3 days/week, with no more than 2 consecutive days without activity. Shorter durations (minimum 75 min/week) of vigorous- intensity or interval training may be sufficient for younger and more physically fit individuals’. Exercise programs were organized by evaluating probable negative effects and contraindications. These physical activities were explained to the patients at the first visit. In the control visits, whether physical activities were performed or not was recorded according to the patient’s declaration.

Patients had medical treatments individually determined. All patients included in the study used metformin. Patients with hyperglycemia symptoms, random plasma glucose > 300 mg/dL or HbA1c 85.3 mmol/mol (> 10 %) were additionally treated with one insulin choice. ADA guidelines suggest that the HbA1C goal for many nonpregnant adults of, 53 mmol/mol (7%) is appropriate. Our patient group was an adult population with no microvascular or macrovascular complications or extensive comorbid conditions. Therefore, our HbA1c goal was 53 mmol/mol (7%) If target HbA1c levels were not reached, a second or third oral antidiabetic (OAD; sulphonylurea/glycine, dipeptidyl peptidase 4 inhibitor and sodium-glucose co-transporter-2) or another insulin choice was added to treatment. Proliferator-activated receptor (PPAR) γ exerts important effects on endotrophin by reducing the transcription of collagen VI molecules [12–14]. Therefore, thiazolidinediones acting on these receptors may affect the results, so no patients were started and patients who were using them were excluded from the study. In our study, which included patients with poor blood glucose regulation, most of the patients were using insulin. In order to reach the target blood glucose new basal insulin treatment was initiated in 4 patients and intensive insulin therapy was added in 9 patients who were already using basal insulin. The SGLT2 inhibitor was started because of its positive effects on weight loss in eligible patients, as suggested by the new diabetes guidelines [11].

### Serum endotrophin measurements

All stored blood samples were thawed only once and on the analysis day. An endotrophin/Pro-C6 ELISA, targeting the C-terminus of the α3 chain of endotrophin, was previously designed (14). Serum endotrophin levels were measured using an enzyme-linked immunosorbent assay (ELISA) method; human endotrophin ELISA kits (Sunred Biological Technology Catalogue No:201-12-9305) were used. The analytical (linear) detection range was 1.5 ng/mL-300 ng/mL, the minimal detection limit was 1.398 ng/mL, the reported intraassay and interassay CV’s were 10 % and 12 %, respectively, for the endotrophin assay kit.

### Follow‐up

Patients were monitored for 3 months. At the end of the third month, height, weight and blood pressure were measured and laboratory tests from the first visit were repeated.

Patients checked blood glucose levels 5 times per day and attended weekly clinical check-ups until blood glucose was regulated.

### Statistical analysis

Statistical analyses used the IBM SPSS statistics for Macintosh. Version 21.0 (Armonk, NY:IBM Corp.). Categoric data are given as frequency. Continuous variables with normal or curve-distribution are given as mean + standard deviation or median (minimum-maximum). Continuous variables were compared with the Student t or Mann Whitney U tests based on distribution. For correlation analysis, the Pearson or Spearman tests were used as required. Each variable had percentage variation values (δ) calculated for initial and 3-month values (δ = new value/old value-1*100). Factors with correlations to δ-endotrophin levels underwent univariate correlation and multiple linear regression analysis to reveal independent correlations with δ-endotrophin levels. Statistical significance was taken as p < 0.05.

## Results

The data from a total of 42 patients who abided by the study criteria were statistically evaluated. Of patients, 23 were female (54.8 %) and 19 were male (45.2 %). Mean age was 55.2 years, with mean diabetes duration of onset 8.14 ± 5.35 years. The demographic and laboratory features of patients included in the study are listed in Table 1. Categorical variables are expressed as number (percentage).

**Table 1:** Baseline characteristics of the study group

Age, (years)	55.16 ± 8.56
Duration of diabetes (years)	8.14 ± 5.35
Women, n (%)	23 (54.8)
Men, n (%)	19 (45.2)
Hypertension^a^, n (%)	26 (61.9)
Retinopathy, n (%)	12 (28.6)
Neuropathy, n (%)	15 (35.7)
Proteinuria^b^, n (%)	19 (45.2)
Body Mass Index^c^ (kg/m²)	29.37 ± 4.6
HbA1c^d^(%)	10.90 ± 1.71

^a^Hypertension; systolic blood pressure > 140 mm / Hg, diastolic blood pressure > 90 mm/Hg

^b^Proteinuria, UACR (urinary albumin creatinine ratio) > 30 mg/g

^c^Body Mass Index; 18–25: normal, 25–30: overweight,> 30: obes*e*

^d^HbA1c ≤ 7 % regulated diabetes, HbA1c > 7 % poorly controlled diabetes

Data are expressed as mean ± standard deviation. Medications used by patients during the study are given in Table 2.

**Table 2:** Baseline treatment of patients

	Treatment at admission	Third month treatment
Metformin, n (%)	42 (100)	42 (100)
DPP-4 inhibitors, n (%)	22 (52.4)	28 (66)
Sulphonylurea, n (%)	10 (23.8)	7 (16.6)
SGLT- 2 inhibitors, n (%)	9 (21.4)	21 (50)
Glinide, n (%)	2 (4.8)	1 (2.3)
Basal insulin, n (%)	36 (85.7)	40 (95)
Regular Insulin, n (%)	22 (52.4)	31 (73.8)

*DPP-4* dipeptidyl peptidase-4, *SGLT* Sodium-glucose co-transporter-2, Glinide (meglitinide)

The laboratory values for patients at first visit and 3 months later are listed in Table 3. After 3 months follow-up, HbA1c, fasting glucose, CRP, UACR and endotrophin levels were observed to clearly reduce (Table 3). Additionally, there were no significant changes observed in BMI, HDL, LDL and TG levels.

**Table 3:** Patient outcomes at baseline and the end of 3 months

	Normal ranges	Baseline n = 42	Third Month n = 42	p value
Endotrophin	–	15.35 ± 9.77	12.20 ± 6.49	0.010
HbA1c (%)	≤ 7	10.90 ± 1.71	6.64 ± 0.36	< 0.001
Fasting glucose (mg/dL)	< 104	258.97 ± 93.55	125.97 ± 14.63	< 0.001
CRP (mg/L)	0–5	6.01 ± 4.24	4.60 ± 3.69	0.050
BMI (kg/m^2^)	18–25	29.37 ± 4.64	29.29 ± 4.41	0.412
HDL, mg/dL	> 50	43.92 ± 8.96	44.14 ± 8.38	0.865
LDL, mg/dL	< 130	144.14 ± 32.64	136 ± 69 ± 30.82	0.160
TG, mg/dL	< 150	197.97 ± 100.22	180.66 ± 78.71	0.208
UACR, mg/g	0–30	15.35 ± 16.04	10.80 ± 9.75	< 0.001

*BMI *body mass index*, HbA1c *hemoglobin A1c,* CRP, *C-reactive protein,* HDL *high-density lipoprotein cholesterol,* LDL *low-density lipoprotein cholesterol,* TG *triglyceride,* UACR *urinary albumin to creatinine(mg/g) ratio

**Fig. 1:**
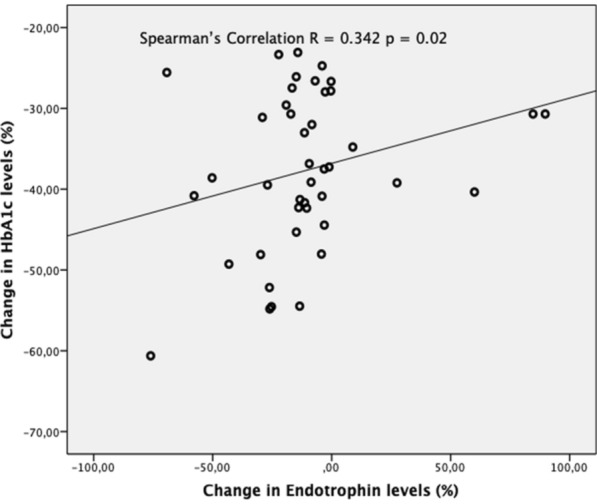
Correlation between changes in serum endotrophin and HbA1c in percentage. Changes in serum endotrophin (δ-endotrophin) and serum HbA1c (δ-HbA1c) in percentage, calculate with values before and after treatment. There is a positive correlation between δ-endotrophin and δ-HbA1c. It means, as HbA1c decreases, endotrophin level decreases

**Fig. 2:**
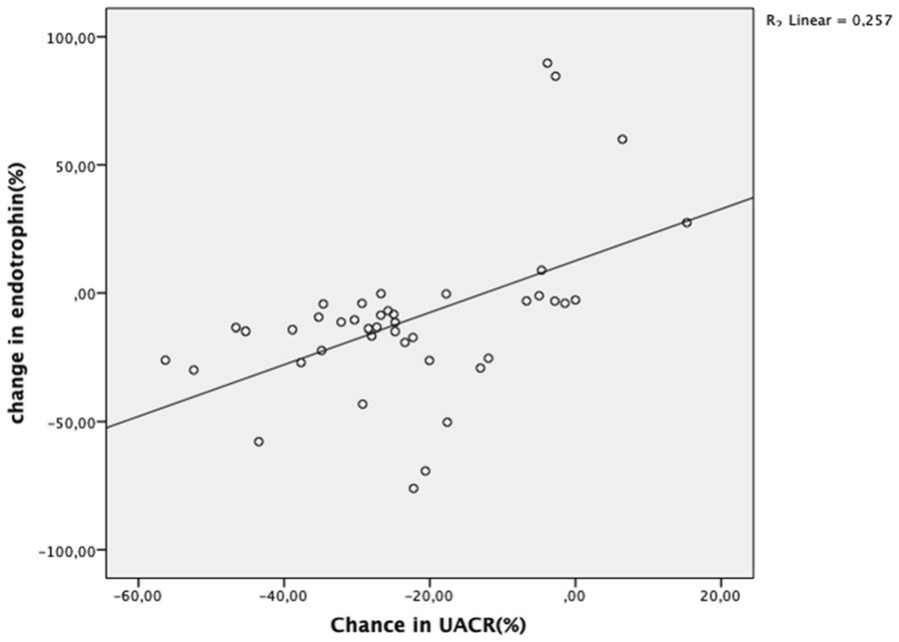
Correlation between changes in serum endotrophin and UACR in percentage. Changes in serum endotrophin (δ-endotrophin) and serum UACR (δ-UACR) in percentage, calculate with values before and after treatment. There is a strong positive correlation between δ-endotrophin and UACR. It means, as UACR decreases, endotrophin level decreases

The correlation coefficients for % change rates (δ) for serum endotrophin levels and other laboratory parameters are shown in Table 4. The variation in serum endotrophin levels examined at the start of the study and in the 3rd month was identified to have a positive correlation with the variation in HbA1c and UACR levels (r = 0.342, p = 0.02; r = 0.484, p = 0.001) (Figs. 1 and 2).

Multiple linear regression analysis showed δ-endotrophin levels were only independently correlated with δ-UACR (model r^2^ = 0.257, p value = 0.00) (Table 4). The δ-HbA1c similarly appeared to have a tendency for independent correlation with δ-endotrophin; however, the correlation was not significant.

**Table 4:** Correlation and regression for -endotrophin

	r	p	OR (95 %Cl)	p
	Univariate correlation		Multivariate regression	
δ-HbA1c	0.342	0.02	0.925 (− 0.034–1.88)	0.058
δ-FBG	0.089	0.50	–	–
δ-BMI	0.190	0.43	–	–
δ-CRP	− 0.019	0.82	–	–
δ-TG	− 0.109	0.49	–	–
δ-HDL-C	0.040	0.80	–	–
δ-LDL-C	0.064	0.343	–	–
δ-UACR	0.484	0.001	1.009 (0.461–1.557)	0.001

*δ-endotrophin = 12.58 +(1.01. δ-UACR)*

### Follow‐up

During follow-up, 2 patients developed hypoglycemia. None of these hypoglycemic events required parenteral glucose infusion or glucagon injection. All patients recovered. Insulin doses were titrated and no patient experienced repeated hypoglycemia.

## Discussion

Diabetes mellitus with poor glycemic control increases risk of death due to endothelial dysfunction, increased inflammatory activity and albuminuria [15]. This study treated T2D patients with poor glycemic control and investigated changes in serum endotrophin levels after target HbA1c levels were reached for the first time.

In our study, significant falls were observed in endotrophin, HbA1c, FBG, CRP and UACR in T2D patients with poor control of glycemic regulation after 3 months of lifestyle changes and medical treatment. The reduction in endotrophin levels was found to be associated with HbA1c and UACR.

In addition to the lipid storage capacity of adipose tissue, it is a very active endocrine and metabolic organ. Many studies have shown adipokines (leptin, adiponectin, resistin, etc.) play an important role in insulin resistance and T2D development.

Endotrophin is a Col 6 subunit secreted by adipose tissue. There are studies showing a positive correlation between Col 6 levels and insulin resistance [16, 17]. However, there is still insufficient data about the effects of Col 6 and endotrophin on glucose metabolism in patients with T2D. A study by Rodriguez et al. found Col 6 and endotrophin causes a dose-linked increase in glucose uptake by muscle cells. This effect was shown to be mediated by integrin receptors [18].

Pasarica et al. in a study of adipose tissue biopsy in obese groups with diabetes and without diabetes found no difference in COL6A3 (endotrophin) levels; and, they stated that COL6A3 did not contribute to diabetes development. They concluded that these results were associated with insulin resistance in both obese groups, but that beta cell injury in obese cases with T2D was additionally not associated with COL6A3 [19]. However, this study did not state HbA1c levels. Additionally, the blood glucose levels of patient group in the study had regulated and were close to levels in the control group.

The oxygen free radicals caused by glucotoxicity have been shown to increase TGF-β synthesis. Collagen VI (endotrophin) has an active role in various biological processes such as inflammation, angiogenesis, fibrosis and epithelial-mesenchymal transition (EMT) by increasing TGF-β synthesis just like glycotoxicity [20, 21]. However, there is insufficient data on the direct effect of glucose on endotrophin metabolis. In an invitro study conducted by Pölivi muona et al. with an electron microscope at the cellular level, it was concluded that “high glucose concentrations increase collogen VI concentration in many cell types”. At high glucose concentrations, it was determined that fibrillar structures were formed in the ECM around the adipose tissue and this impaired microenvironment induced collagen VI increase. Macrophage and fibroblast suppression can be achieved by regulation of blood glucose. This creates a more stable microenvironment. Therefore, it is expected that the concentration of endotrophin will decrease in good glycemic regulation [17].

In our study, we included patients with poorly controlled T2D and HbA1c levels of 74.9 mmol/mol (9 %) and above. In this way, we looked at the relationship between serum endotrophin levels and diabetes regulation. Our study data shown that endotrophin levels were initially high in T2D patients with poor glycemic control, but this significantly reduced after treatment. We also found a positive correlation between endotrophin and HbA1c levels. We think low insulin reserves in the pancreas and resulting poor blood glucose control disrupts adipose tissue functions creating a negative effect on endotrophin levels, together with obesity. We assumed that despite the catabolic effects of poor glycemic control in patients included in our study, weight loss could not be achieved despite the appropriate diet due to anabolic effects caused by glycemic control and insulin use. In conclusion, there was no significant change in BMI in our overweight and obese patients over a 3-month period. The lack of no weight loss allowed us to evaluate the parameters that changed after blood glucose regulation independently of weight. We thought that blood glucose control may be responsible for the decrease in endotrophin levels, independent of BMI.

Some studies have reported moderate-degrees of increase in the inflammation marker of CRP predicts the development of insulin resistance [22–24]. Additionally, Tanigaki et al. showed moderate elevations in CRP caused insulin resistance in mice [25]. When we examine the inflammation marker of CRP in our study, as expected, the CRP levels reduced along with glycemic regulation. The reduction in serum endotrophin levels with blood glucose regulation and similar reduction in CRP levels leads to the consideration that there was a positive effect on inflammation.

Endotrophin increases the synthesis of TGF-β and regulates insulin gene transcription via the SMAD3 pathway of the TGF-β signal. TGF-β superfamily expression levels were shown to control blood glucose levels [20, 21]. As is known, TGF-β is associated with diabetic cardiomyopathy [26] and diabetic nephropathy [27] development. For this reason, studies about the TGF-β signal pathway as a treatment modality to reduce islet cell inflammation and preserve beta cell differentiation have gained importance [21]. Endotrophin increasing TGF- β synthesis are worth investigating for their effect on diabetes and complications in this sense.

Endotrophin is an adipokine with a basic role in fibrosis and inflammation. At the same time, it is one of the most commonly found proteins in glomerular ECM and increases in renal injury [28, 29]. Renal fibrosis is known to be the basic pathologic route in chronic renal injury. Fenton et al. [30] in a 500-cohort study found a significant inverse correlation between EGFR and serum endotrophin levels. Here they stated the increase in Col-6 and endotrophin may be explained by increased fibrotic tissue production with renal injury. The same study found high endotrophin levels caused an increase in the progression of end stage renal failure and found it was associated with increased mortality [30]. In accordance with this data, our patient group with poor glycemic control were identified to have a significant reduction in UACR with blood glucose regulation. Additionally, we identified a positive correlation between the reduction in UACR and endotrophin levels. Regression analysis found the δ-UACR value significantly explained the δ-endotrophin level. This means that if there is greater variation in UACR, the variation in endotrophin levels will be similarly high, and if the variation in UACR is low, the variation in endotrophin levels in patients will be minimal. Additionally, this variation is independent of HbA1c variation and hence independent of blood glucose. These results lead to the consideration that there is a tight and independent correlation between endotrophin and the microalbuminuria, one of the microvascular complications of diabetes and we think that studies targeting endotrophin will contribute to preventing the progression of diabetic nephropathy in the future.

Limitations of our study include the relatively small sample and short follow-up duration. And the other limitation of this study is; there is not enough data in the literature about how other antidiabetic agents affect endorphin levels. This study dealt with the correlation of endotrophin levels in T2D and is the first study to research the longitudinal correlation between endotrophin levels, glycemic control and microalbuminuria.

## Conclusions

For patients with T2D, lifestyle changes and appropriate medical treatment can provide blood glucose regulation. Endotrophin levels decreased significantly with the decrease in HbA1c and glucose regulation. This reduction in endotrophin levels is closely associated with the reduction in UACR. This relationship was identified to be associated with the variation in endotrophin levels independent of blood glucose regulation. Advanced studies with long-term guideline-based treatment should be performed to determine the full role of endotrophin with prognostic value for patients with T2D.

## Data Availability

Upon request from the corresponding author, data can be provided.
